# Shotgun Metagenomics Reveals Gut Microbiome Remodeling with Altered Taxonomic Composition and Functional Potential in Diabetic Dogs

**DOI:** 10.3390/ani16060936

**Published:** 2026-03-16

**Authors:** Qi An, Siyu Chen, Shizhen Ma, Rina Bai, Zijie Lu, Yang Liu, Fan Wang, Qian Wang, Yu Song, Gege Zhang, Yanli Lyu, Lu Wang, Yang Wang, Zhaofei Xia

**Affiliations:** 1 College of Veterinary Medicine, China Agricultural University, Beijing 100193, China; anqicau@163.com (Q.A.); siyuchen@cau.edu.cn (S.C.); bairina@163.com (R.B.); wangfan1112024@163.com (F.W.); wq15797861626@gmail.com (Q.W.); sy1296328095@126.com (Y.S.); 13051557166@163.com (G.Z.); ly_annie@163.com (Y.L.); 2 Veterinary Teaching Hospital, China Agricultural University, Beijing 100193, China; alloblue@aliyun.com (Z.L.); luyanli@cau.edu.cn (Y.L.); 3 State Key Laboratory for Diagnosis and Treatment of Severe Zoonotic Infectious Diseases, Key Laboratory for Zoonosis Research of the Ministry of Education, College of Veterinary Medicine, Jilin University, Changchun 130015, China; mashizhen@jlu.edu.cn; 4 Key Laboratory of Animal Antimicrobial Resistance Surveillance, Ministry of Agriculture and Rural Afairs, China Agricultural University, Beijing 100193, China; lwang@cau.edu.cn; 5 State Key Laboratory of Veterinary Public Health and Safety, College of Veterinary Medicine, China Agricultural University, Beijing 100193, China

**Keywords:** fecal microbiome, shotgun metagenomic sequencing, diabetes mellitus, canine

## Abstract

Gut microbiota dysbiosis is implicated in metabolic disorders, yet taxonomic and functional alterations in canine diabetes remain incompletely defined. Here, we performed shotgun metagenomic sequencing of fecal samples from 38 diabetic dogs and 37 healthy controls under controlled conditions (no recent antibiotic/probiotic exposure and stable commercial diets). Alpha-diversity indices did not differ between groups, whereas beta-diversity revealed significant separation of community structure at both genus and species levels (*p* < 0.05). Linear discriminant analysis effect size (LEfSe) identified enrichment of opportunistic-associated taxa in diabetic dogs, including Enterobacterales/Enterobacteriaceae (e.g., *Escherichia coli*, *Klebsiella pneumoniae*, *Salmonella enterica*) and *Enterococcus faecalis*. In contrast, healthy dogs were enriched for putatively beneficial taxa linked to bile acid and short-chain fatty acid (SCFA) metabolism, including *Turicibacter* spp. and *Romboutsia* spp. Functional profiling showed higher abundances of pathways related to carbohydrate/energy metabolism, membrane transport, and virulence/colonization in diabetic dogs; 17 KEGG level-3 pathways and 320 KOs differed at FDR < 0.05, with enriched modules including bacterial secretion systems, lipopolysaccharide biosynthesis, chemotaxis/flagellar assembly, and biofilm formation. Collectively, canine diabetes is associated with a remodeled gut microbiome characterized by expansion of opportunistic pathogens and elevated virulence and metabolic potential, supporting exploration of microbiota-targeted strategies as a complement to conventional management.

## Simple Summary

Diabetes is a common metabolic disease in dogs, but how it is related to changes in gut bacteria is still not well understood. In this study, we analyzed stool samples from diabetic and healthy dogs that were kept under similar conditions, including stable diets and no recent use of antibiotics or probiotics. We found that overall bacterial richness was similar between the two groups, but the types of bacteria present were clearly different. Dogs with diabetes had higher levels of bacteria that are often associated with infections, while healthy dogs had more bacteria linked to normal digestion and production of beneficial metabolites. In addition, the gut bacteria of diabetic dogs showed increased genetic potential for energy use, transport of substances, and traits related to bacterial survival and colonization. These results indicate that diabetes in dogs is associated with clear changes in both the composition and potential functions of gut bacteria. Understanding these changes may help develop new strategies to support diabetes management through improving gut health.

## 1. Introduction

Diabetes mellitus is a common endocrine disease in dogs. Epidemiological studies have reported that the prevalence of canine diabetes mellitus (DM) ranges from 0.26% to 1.33% in the United Kingdom, the United States, Sweden, and Italy [1,2,3,4], and this rate has shown a continuous upward trend over time [5,6]. The diagnosis of canine diabetes is primarily based on persistent hyperglycemia and is typically accompanied by characteristic clinical manifestations, including glucosuria, polyuria, polydipsia, polyphagia, and weight loss [7]. At present, the precise mechanisms underlying hyperglycemia in dogs have not been fully elucidated. Available evidence indicates that canine diabetes shares pathological similarities with human type 1 diabetes (T1D). Its main features involve the loss of pancreatic β-cells and insufficient insulin secretion [8]. Therefore, most diabetic dogs require lifelong insulin therapy. This treatment must be combined with strict dietary management and regular exercise to achieve effective glycemic control [9]. In addition, canine diabetes is often accompanied by complications such as cataracts, chronic pancreatitis, and bacterial infections, imposing substantial lifestyle and economic burdens on pet owners.

In recent years, studies have demonstrated that the gut microbiota plays a key role in the development and progression of metabolic diseases, including DM [10]. Gut microbiota dysbiosis can promote autoimmune destruction of pancreatic endocrine tissue by inducing inflammatory responses and immune dysregulation [11]. Compared with healthy individuals, patients with diabetes exhibit significant differences in gut microbiota composition [12]. Changes in the gut microbiota composition of patients with T1D have been shown to be associated with blood glucose control and diabetes-related complications, suggesting that the gut microbiota may also be involved in the development and progression of diabetes-related complications [13]. Moreover, mice receiving fecal microbiota transplants from diabetic patients develop early diabetic phenotypes. Early interventions targeting the gut microbiome can delay disease progression in T1D mouse models [14,15]. Collectively, these findings highlight the important role of the gut microbiota in the pathogenesis of human diabetes. Despite the accumulation of substantial evidence in human medicine, the role of the gut microbiota in canine diabetes remains relatively underexplored. Current studies on the gut microbiota of diabetic dogs have mainly relied on 16S rRNA gene sequencing and sample sizes have been limited, making it difficult to comprehensively characterize microbial composition and functional features [16,17,18].

With advances in high-throughput sequencing technologies, shotgun metagenomic sequencing has been widely applied in microbiome research due to its ability to provide higher taxonomic resolution, more comprehensive diversity information, and more accurate prediction of functional genes [19]. To more systematically elucidate the association between canine diabetes and gut microbial functional characteristics, this study designed a clinically relevant case–control cohort with an expanded sample size to improve statistical robustness and implemented stricter control of major microbiome-modifying exposures, including recent antibiotic and probiotic use and diet standardization (restricted to commercial dog food without probiotic supplementation). Shotgun metagenomics was then used to enable higher-resolution taxonomic profiling and to characterize the microbiome’s functional potential in diabetic versus healthy dogs. By integrating fine-scale taxonomic shifts with changes in functional potential, this study aims to provide new insights into microbiota-associated functional-potential alterations in diabetic dogs and to improve understanding of potential microbiological mechanisms underlying canine diabetes.

## 2. Materials and Methods

### 2.1. Study Design

Fecal samples were collected from dogs in Beijing, Tianjin, and Hebei, China, between June 2023 and September 2025. The cohort included 38 dogs with DM and 37 healthy control dogs. Dogs in the diabetic group were enrolled if they met at least two of the following three diagnostic criteria:

1. Presence of typical clinical signs, including polyuria and polydipsia;
2. A positive urine glucose test;
3. Evidence of persistent hyperglycemia, defined as glycated hemoglobin or fructosamine levels above the normal reference range, or fasting blood glucose concentrations exceeding the normal reference range on at least two separate occasions.

If renal glycosuria could not be excluded, the criterion of persistent hyperglycemia was required for inclusion.

Healthy dogs were enrolled based on the absence of apparent abnormalities in blood biochemical parameters and the lack of clinical or laboratory evidence of infectious or metabolic diseases. To minimize the potential effects of diet and medication on the gut microbiota, dogs in both the diabetic and healthy groups were required to meet the following conditions:

1. Consumption of a commercial dog food (Supplementary Table S1) without probiotic supplementation, with no changes in diet within two weeks prior to sample collection;
2. No administration of antibiotics or probiotic preparations within one month prior to enrollment;
3. Absence of concurrent diseases including gastrointestinal infections, inflammatory bowel disease, or gastrointestinal tumors that could markedly alter the gut microbiota.

### 2.2. Sample Collection

Fresh fecal samples were collected immediately after natural defecation from each enrolled dog using sterile, disposable containers (Thermo Fisher Scientific, Waltham, MA, USA). The samples were then transported to the laboratory under low-temperature conditions. Under aseptic conditions in a laminar flow hood (Esco, Singapore), the inner portion of the fecal material that had not been exposed to environmental surfaces was transferred into sterile centrifuge tubes (Thermo Fisher Scientific, Waltham, MA, USA). Samples were then snap-frozen in liquid nitrogen and immediately stored at −80 °C until subsequent DNA extraction and metagenomic sequencing analyzes.

### 2.3. DNA Extraction and Metagenomic Sequencing

Total microbial genomic DNA samples were extracted using the OMEGA Mag-Bind Soil DNA Kit (Omega Bio-Tek, Norcross, GA, USA), following the manufacturer’s instructions, and stored at −20 °C prior to further assessment. The quantity and quality of extracted DNA were measured using a Qubit™ 4 Fluorometer (Invitrogen, Carlsbad, CA, USA) and agarose gel electrophoresis, respectively. The extracted microbial DNA was processed to construct metagenome shotgun sequencing libraries with insert sizes of ~400 bp by using Illumina TruSeq Nano DNA LT Library Preparation Kit (Illumina, San Diego, CA, USA) [20]. Each library was sequenced by Illumina NovaSeq platform (Illumina, San Diego, CA, USA) with PE150 strategy at Personal Biotechnology Co., Ltd. (Shanghai, China).

### 2.4. Metagenomics Analysis

Raw sequencing reads were processed to obtain high-quality reads for downstream analyzes. First, sequencing adapters were removed using Cutadapt (v1.2.1) [21]. Low-quality reads were then trimmed using a sliding-window algorithm implemented in fastp (v0.23.2) [22]. Subsequently, reads were aligned to the canine reference genome using Minimap2 (v2.24-r1122) [23] to remove host-derived sequences. Clean reads were then assembled de novo for each sample using MEGAHIT (v1.1.2) [24]. Contigs longer than 300 bp were pooled and dereplicated by sequence clustering using MMseqs2 (v13.45111), with a sequence identity threshold of 0.95 and a minimum coverage of 90% for the shorter contig. Taxonomic annotations of gene sequences across different ranks (domain, phylum, class, order, family, genus, and species) were generated using the MMSeqs2-based 2bLCA algorithm [25]. Contigs assigned to non-bacterial taxa (e.g., archaea, viruses, and other non-Bacteria assignments) were removed prior to downstream compositional and differential abundance analyses; only contigs classified as bacteria were retained. Gene prediction was performed on the non-redundant contigs using Prodigal (v2.6.3) [26]. Finally, non-redundant gene sequences were annotated by searching against the KEGG protein database using MMseqs2 to obtain functional annotations.

### 2.5. Statistical Analysis

Microbial community composition was analyzed based on assembled contigs. The abundance of each taxon was calculated within each group and averaged, and each group was then treated as a whole for compositional analysis. Based on the taxonomic and functional profiles of non-redundant genes, Linear discriminant analysis effect size (LEfSe) was performed to detect differentially abundant taxa and functions across groups using the default parameters. β-diversity analysis was performed to investigate the compositional and functional variation of microbial communities across samples using Bray–Curtis distance metrics and visualized via principal coordinate analysis (PCoA).

## 3. Results

### 3.1. Basic Information of the Enrolled Dogs

A total of 38 dogs with DM and 37 healthy dogs were included in this study following strict diagnosis and screening according to the predefined inclusion criteria (Table 1). The mean age of diabetic dogs was 10 years, with a median age of 10 years, whereas the healthy dogs had a mean age of 9 years and a median age of 8 years. The age difference between the two groups was statistically significant (*p* < 0.05). All enrolled dogs were > 7 years and classified as senior according to the 2019 AAHA Canine Life Stage Guidelines [9].

Among the diabetic dogs, 12 (31.6%) were intact males, 4 (10.5%) were neutered males, 13 (34.2%) were intact females, and 9 (23.7%) were spayed females. Among the healthy dogs, 20 (54.1%) were intact males, 3 (8.1%) were neutered males, 8 (21.6%) were intact females, and 6 (16.2%) were spayed females. The study population was predominantly composed of small companion breeds. Poodles were the most prevalent breed, accounting for 22 (57.9%) dogs in the diabetic group and 18 (48.6%) dogs in the healthy group. Mixed-breed dogs were the second most common, with 2 (5.3%) in the diabetic group and 4 (10.8%) in the healthy group. The remaining breeds were distributed sparsely across both groups. The blood glucose level in the diabetic group, with a median of 429.9 (378.2, 500.7), was significantly higher than that in the healthy group, which had a median of 98.3 (90.7, 103.0).

### 3.2. Metagenomic Data

After removal of host-derived sequences and dereplication of the predicted genes, a gene catalog comprising 3,450,163 non-redundant protein-coding genes (ORFs) was constructed. The total length of the gene catalog was 1.91 Gbp, with an average gene length of 554.6 bp. Based on the non-redundant gene catalog, taxonomic and functional annotations were performed. Bacterial taxonomic annotation identified 108 phyla, 183 classes, 328 orders, 668 families, 2462 genera, and 11,718 species. Functional annotation using the KEGG database identified 6 level-1 (KEGG L1), 45 level-2 (L2), and 390 level-3 (L3) functional pathways, as well as 13,139 KEGG Orthology (KO) functional units.

### 3.3. Characteristics of Gut Microbiota Composition in Diabetic and Healthy Dogs

**Figure 1:**
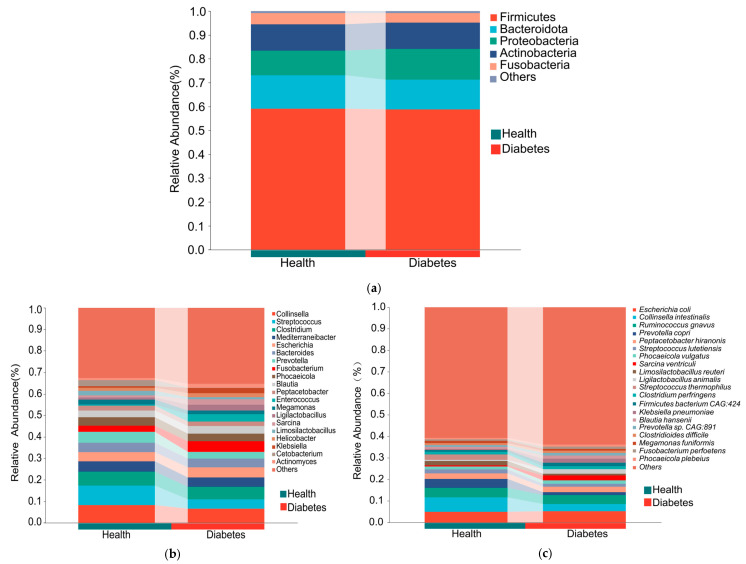
Group-averaged gut microbiota composition in diabetic and healthy dogs. (**a**) Phylum-level composition of gut microbiota, showing only the top 5 most abundant bacterial phyla with the remaining taxa grouped as “Others”. (**b**) Genus-level composition of gut microbiota, showing only the top 20 most abundant bacterial genera, with the remaining taxa grouped as “Others”. (**c**) Species-level composition of gut microbiota, showing only the top 20 most abundant bacterial species, with the remaining taxa grouped as “Others”.

At the phylum level, the gut microbiota of diabetic dogs was mainly composed of Firmicutes (59.0%), Bacteroidota (14.1%), Actinobacteria (11.0%), and Proteobacteria (10.3%). Similarly, the gut microbiota of healthy dogs was dominated by Firmicutes (58.9%), followed by Proteobacteria (12.9%), Bacteroidota (12.4%), and Actinobacteria (11.0%) (Figure 1a). Overall, the two groups exhibited a largely comparable microbial composition at the phylum level. At the genus level, the most abundant genera in diabetic dogs included *Collinsella* (6.5%), *Clostridium* (5.8%), *Fusobacterium* (5.0%), *Escherichia* (4.6%), and *Streptococcus* (4.4%). The dominant genera in healthy dogs were *Streptococcus* (9.1%), *Collinsella* (8.3%), *Clostridium* (6.4%), *Prevotella* (5.1%), and *Mediterraneibacter* (4.8%) (Figure 1b). At the species level, the predominant species in diabetic dogs were *E. coli* (5.2%), *Ruminococcus gnavus* (4.2%), *Collinsella intestinalis* (3.3%), *Peptacetobacter hiranonis* (2.6%), and *Sarcina ventriculi* (2.3%). In healthy dogs, the most abundant species included *C. intestinalis* (6.7%), *E. coli* (4.9%), *R. gnavus* (4.4%), *Prevotella copri* (4.1%), and *Streptococcus thermophilus* (2.6%) (Figure 1c).

### 3.4. Comparison of Gut Microbiota Diversity Between Diabetic and Healthy Dogs

**Figure 2:**
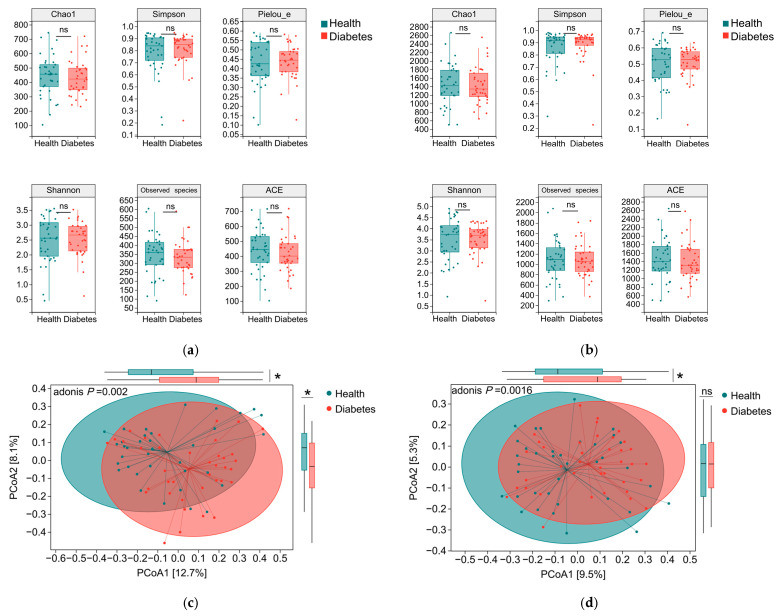
Comparison of gut microbiota diversity between diabetic and healthy dogs. (**a**) α-diversity of the gut microbiota at the genus (**a**) and species (**b**) level, including the Chao1, ACE, observed species, Shannon, Simpson, and Pielou’s evenness (Pielou_e) indices. Principal coordinates analysis (PCoA) of Bray–Curtis dissimilarities showing gut microbiota β-diversity between diabetic and healthy dogs at the genus (**c**) and species (**d**) levels. Group differences were assessed using PERMANOVA (adonis2) with adjustment for age, sex, and neuter status (9999 permutations). Differences between diabetic and healthy dogs were assessed using the Wilcoxon rank-sum test. * *p* < 0.05; ns, not significant.

No statistically significant differences were observed in any α-diversity indices between the diabetic and healthy dog groups, including indices of species richness (Observed species, Chao1, and ACE), diversity (Shannon and Simpson), and community evenness Pielou_e (*p* > 0.05; Figure 2a,b). This indicates that, under the conditions of this study, no significant differences were observed between diabetic and healthy dogs in terms of within-sample gut microbial richness, evenness, or overall diversity. Accordingly, the two groups exhibited largely comparable within-sample microbial complexity and abundance distributions. On the basis of comparable within-sample diversity, differences in the overall composition and structure of the gut microbiota between diabetic and healthy dogs were further examined using β-diversity analysis based on Bray–Curtis dissimilarity. Principal coordinate analysis (PCoA) was used to visualize the β-diversity patterns and revealed a discernible separation between the two groups at both the genus and species levels (Figure 2c,d). Group differences were evaluated using PERMANOVA (adonis2) on Bray–Curtis dissimilarities while adjusting for age, sex, and neuter status (9999 permutations). After covariate adjustment, β-diversity remained significantly different between diabetic and healthy dogs at both the genus (R^2^ = 0.0295, *p* = 0.0006) and species (R^2^ = 0.0229, *p* = 0.0016) levels; however, the effect sizes were small (R^2^ ≈ 0.02–0.03), indicating limited variance explained by group status. The distribution of pairwise Bray–Curtis dissimilarities (within-group vs. between-group) is provided in Supplementary Figures S1 and S2. We additionally confirmed that the PERMANOVA results were not attributable to differences in within-group dispersion (PERMDISP: genus *p* = 0.296; species *p* = 0.218).

### 3.5. Differentially Abundant Gut Microbiota Taxa Between Diabetic and Healthy Dogs

**Figure 3:**
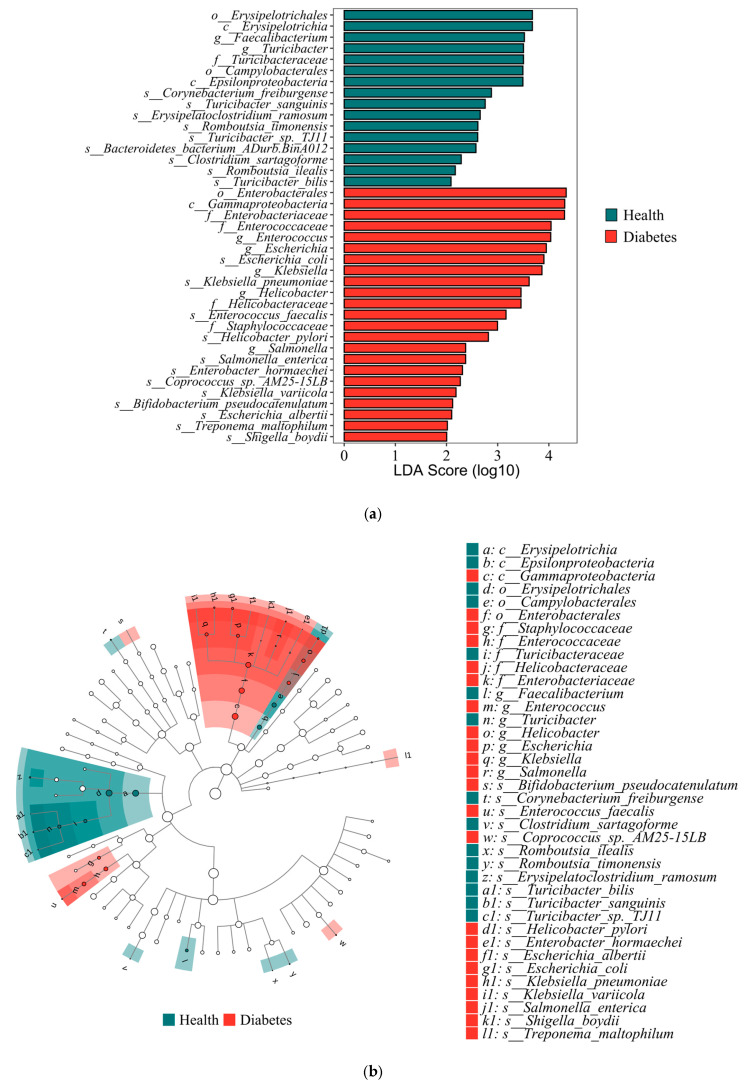
The gut microbiota differences between diabetic and healthy dogs were identified by Linear discriminant analysis effect size (LEfSe) analysis. The thresholds were set at *p* < 0.05 and linear discriminant analysis (LDA) score > 2. (**a**) Bar plot showing taxa with significant differences identified by LEfSe (|LDA score| > 2). (**b**) Cladogram illustrating the phylogenetic distribution of differentially abundant taxa.

**Figure 4:**
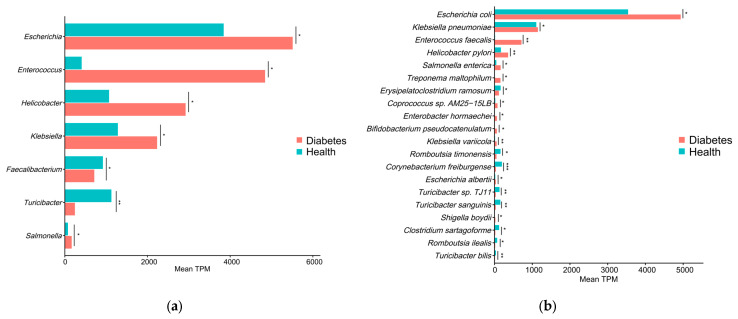
Mean abundance (Transcripts Per Million, TPM) of enriched taxa at the genus (**a**) and species (**b**) levels in diabetic and healthy dogs. * *p* < 0.05, ** *p* < 0.01, *** *p* < 0.001.

LEfSe analysis was performed to characterize differential features of the gut microbiota between diabetic and healthy dogs. This method combines the Wilcoxon rank-sum test with linear discriminant analysis (LDA) to identify microbial taxa that best explain differences between groups. Taxa with *p* < 0.05 and LDA scores >2.0 were considered significantly discriminative features. A total of 38 microbial taxa across different taxonomic levels were identified as significantly different between diabetic and healthy dogs (Figure 3a). These taxa constitute a set of potential microbial biomarkers that distinguish the two groups. For instance, *Enterococcus*, *Escherichia*, *Klebsiella*, and *E. coli* (LDA > 3) were significantly enriched in diabetic dogs, whilst *Faecalibacterium* and *Turicibacter* (LDA > 3) were enriched in healthy dogs. To further visualize the phylogenetic distribution of differentially abundant taxa, a taxonomic cladogram was generated and revealed that taxa enriched in diabetic dogs were mainly clustered within two major phylogenetic lineages (Figure 3b). The first lineage belonged to the class Gammaproteobacteria. It included members of the order Enterobacterales and the family Enterobacteriaceae, as well as the genera *Salmonella* (diabetes vs. health: 165.2 vs. 72.6), *Escherichia* (5512.3 vs. 3842.3), and *Klebsiella* (2232.3 vs. 1281.2), including the species *Salmonella enterica* (161.2 vs. 47.1), *E. coli* (4935.1 vs. 3540.5), and *Klebsiella pneumoniae* (1150.0 vs. 1106.0). The second lineage comprised taxa from the families Staphylococcaceae and Enterococcaceae, represented primarily by the genus *Enterococcus* (4843.2 vs. 406.7) and the species *Enterococcus faecalis* (714.8 vs. 14.0). In addition, contigs annotated as *Helicobacter pylori* (361.5 vs. 168.2) were also significantly enriched in the gut microbiota of diabetic dogs. Other taxa enriched in the diabetic group included *Treponema maltophilum*, *Enterobacter hormaechei*, *Bifidobacterium pseudocatenulatum*, *Klebsiella variicola*, *Escherichia albertii*, and *Shigella boydii*. Conversely, taxa enriched in healthy dogs were mainly associated with the class Erysipelotrichia, including members of the order Erysipelotrichales, family Turicibacteraceae, and genus *Turicibacter* (diabetes vs. health: 243.4 vs. 1124.0), with representative species such as *Turicibacter bilis* (13.3 vs. 35.3) and *Turicibacter sanguinis* (32.7 vs. 152.3). Moreover, species associated with short-chain fatty acids (SCFAs) production, including *Romboutsia ilealis* (19.0 vs. 69.0) and *Romboutsia timonensis* (52.8 vs. 157.4), were also significantly enriched in healthy dogs. Other taxa enriched in the healthy group included *Faecalibacterium*, *Erysipelatoclostridium ramosum*, *Corynebacterium freiburgense* and *Clostridium sartagoforme*. These quantitative comparisons are visualized in Figure 4.

### 3.6. Functional Differences in the Gut Microbiota Between Diabetic and Healthy Dogs

**Figure 5:**
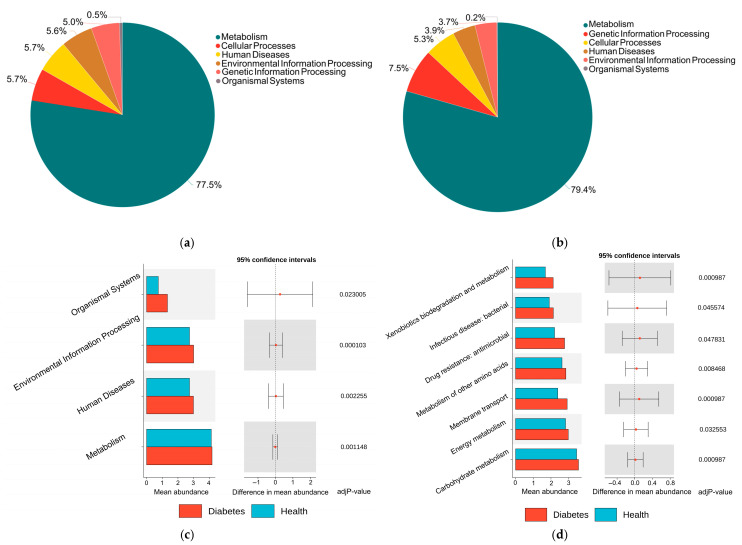
KEGG functional profiles of the gut microbiota in diabetic and healthy dogs. (**a**) KEGG Level 1 functional composition of the gut microbiota in diabetic dogs. (**b**) KEGG Level 1 functional composition of the gut microbiota in healthy dogs. (**c**) Comparison of KEGG Level 1 functional categories between diabetic and healthy dogs. (**d**) Comparison of KEGG Level 2 functional categories between diabetic and healthy dogs. Differences were assessed using the Wilcoxon rank-sum test with Benjamini–Hochberg correction for multiple comparisons, and an FDR < 0.05 was considered statistically significant.

Based on KEGG Level 1 annotation, the functional composition of the gut microbiota in diabetic and healthy dogs was comparatively analyzed. The functional profiles of both groups were dominated by metabolism, accounting for 77.5% (diabetic group) and 79.4% (healthy group), respectively (Figure 5a,b). In the diabetic group, cellular processes (5.7%) and human diseases (5.7%) followed, whereas in the healthy group, genetic information processing (7.5%) and cellular processes (5.3%) were the second and third categories. To further systematically evaluate the functional differences between the two groups, Wilcoxon rank-sum tests were performed on functional abundance at different KEGG annotation levels (Level 1, Level 2, Level 3, and KO level) with multiple comparison correction using the Benjamini-Hochberg method (FDR < 0.05 considered statistically significant). At KEGG Level 1, functions related to metabolism, human diseases, environmental information processing, and organismal systems were significantly higher in the gut microbiota of diabetic dogs compared to healthy dogs (FDR < 0.05; Figure 5c). Within these differential Level 1 categories, several Level 2 functional subclasses were found to be significantly enriched in the diabetic group, including carbohydrate metabolism, energy metabolism, xenobiotics biodegradation and metabolism, and metabolism of other amino acids under metabolism; drug resistance: antimicrobial and infectious disease: bacterial under human diseases; and membrane transport under environmental information processing (FDR < 0.05; Figure 5d). To further investigate functional differences at finer hierarchical levels, we performed differential analyzes of functional abundances at the KEGG Level 3 and KO levels (Supplementary Tables S2 and S3). We identified 17 KEGG Level 3 pathways and 320 KOs that differed significantly between diabetic and healthy dogs (FDR < 0.05), indicating that group differences extend beyond Level 1–2 and were widespread at higher functional resolution.

### 3.7. KEGG Pathway Enrichment Analysis Based on Differential KOs

**Figure 6:**
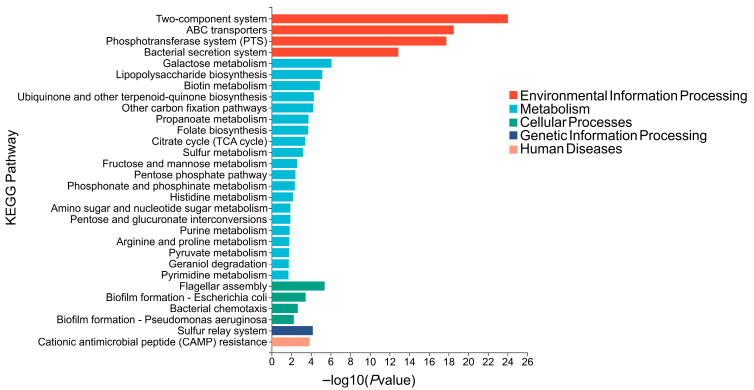
KEGG pathway enrichment of KOs significantly upregulated in diabetic dogs relative to healthy dogs. Differential KOs used for enrichment were defined as *p* < 0.05; |FC| > 1.2. Pathways with *p* < 0.05 were considered significantly enriched.

To summarize the biological pathways represented by the differential abundant KOs and to avoid fragmented interpretation of individual KOs, KEGG enrichment pathways analysis was subsequently performed based on differential KOs. Multiple pathways were significantly enriched among upregulated KOs in diabetic dogs, whereas comparatively few pathways were enriched among downregulated KOs (*p* < 0.05; |FC| > 1.2; Supplementary Table S4). Therefore, we focused on the pathways enriched among KOs significantly upregulated in diabetic dogs relative to healthy dogs (Figure 6). The enriched pathways were predominantly clustered within the Metabolism category, indicating a significant aggregation of differential KOs in metabolic functions. These pathways were mainly involved in central carbon metabolism and carbohydrate utilization, with representative examples including the pentose phosphate pathway, pyruvate metabolism, and the citrate cycle (TCA cycle); nucleotide and cofactor metabolism, such as purine metabolism, pyrimidine metabolism, folate biosynthesis, and biotin metabolism; and the synthesis of cell envelope–related sugar precursors and outer membrane components, including amino sugar and nucleotide sugar metabolism and lipopolysaccharide biosynthesis. Besides metabolism, differential KOs in diabetic dogs were also significantly enriched in several pathways related to environmental information processing and cellular processes. These included two-component system, ABC transporters, phosphotransferase system (PTS), and bacterial secretion system, as well as flagellar assembly, bacterial chemotaxis, and biofilm formation—*E*. *coli*/*Pseudomonas aeruginosa*. Furthermore, the sulfur relay system (genetic information processing) and cationic antimicrobial peptide (CAMP) resistance (human diseases) pathways were also significantly enriched in diabetic dogs.

## 4. Discussion

In this study, we found significant changes in both the composition and functional gene profile of the gut microbiota in diabetic dogs. In diabetic dogs, Enterobacteriaceae members were notably enriched, particularly *Escherichia*, *Klebsiella* and *Salmonella* (including *E. coli*, *S. enterica* and *K. pneumoniae* among others). In the healthy group, *Turicibacter* and bile acid/SCFA-associated taxa such as *R. ilealis* and *R. timonensis* were significantly enriched. Functional analysis further revealed significant enrichment of microbial pathways related to virulence-associated functions, environmental adaptation, and carbohydrate metabolism. Notably, in a relatively large shotgun metagenomic cohort (*n* > 30 per group), this study observed a concordant trend between shifts in microbial community composition and changes in the functional gene profile. The increased abundance of opportunistic-associated taxa may parallel diabetes-associated chronic inflammation and metabolic dysregulation. Moreover, given the increasingly close contact between companion animals and humans [27], surveillance of these opportunistic-associated taxa may also be relevant from a One Health and public health perspective. Our findings therefore underscore the necessity for ongoing surveillance of both the composition and functional potential of the gut microbiota in diabetic dogs and support the exploration of microbiota-targeted interventions. This integrated approach is important for effective disease management as well as for broader public health risk assessment. However, further studies are needed, such as strain-level typing and AMR/virulence profiling, to assess their zoonotic potential and transmission relevance. Specifically, metagenomic results can be comprehensively mapped and annotated against CARD and VFDB to characterize antimicrobial resistance and virulence gene profiles, quantify key AMR determinants and virulence factors, and compare their distributions between groups.

The α-diversity of the gut microbiota did not differ significantly between diabetic and healthy dogs, which aligns with previous canine diabetes studies [16,17,18] and with reports in human T1D cohorts using 16S rRNA sequencing or metagenomics [13,28,29,30,31,32]. The β-diversity analysis revealed a statistically significant separation in the community structure between diabetic dogs and healthy dogs in agreement with prior canine and human diabetes studies [16,29,30,33], although the effect size was limited (R^2^ ≈ 0.02–0.03). Together, these results suggest that diabetes-associated dysbiosis is characterized primarily by community remodeling (shifts in the abundance of specific taxa) rather than a global loss of microbial diversity.

The gut microbiota of diabetic dogs was enriched in multiple opportunistic taxa. Similar patterns have been reported in human diabetes cohorts, including *Escherichia*, *Enterococcus*, *Salmonella* [10], *E. coli*, *K. pneumoniae* [14], and *E. faecalis* [34,35]. In humans, increased *Escherichia* abundance has been associated with infection susceptibility and higher blood glucose [36,37], and *Enterococcus* has been positively correlated with HbA1c in T1D [13,38]. *Salmonella* was significantly enriched in patients with severe diabetes [39]. Mechanistically, evidence from human studies suggests that diabetes-associated chronic inflammation is frequently attributed to Gram-negative LPS exposure [40,41,42]. This inflammatory state is also associated with reduced insulin sensitivity [43]. Under high-glucose and high-LPS conditions, murine experimental studies indicate LPS can activate the NOD1-RICK inflammatory signaling pathway, thereby exacerbating inflammatory responses [44] inducing pancreatic β-cell damage [45]. In addition, several human studies suggest that an increased abundance of intestinal opportunistic-associated taxa may disrupt gut barrier integrity [46,47,48], promote the release of pro-inflammatory mediators [49,50], and contribute to insulin resistance [51,52,53]. Contigs annotated as *H. pylori* were enriched in diabetic dogs; however, given the close relatedness within the genus *Helicobacter* and the resulting risk of species-level misannotation in reference-based classification, this finding is interpreted cautiously and considered a *Helicobacter*-related signal rather than definitive *H. pylori* colonization.

In this study, *Turicibacter* and *Romboutsia*, including species like *T. sanguinis*, *T. bilis*, *R. ilealis*, and *R. timonensis*, were significantly enriched in healthy dogs. In humans, *Turicibacter* and *Romboutsia* have been associated with bile acid metabolism and short-chain fatty acid (SCFA)-related functions. Higher *T. sanguinis* abundance has also been linked to a lower risk of human metabolic disease [54,55]. Additionally, *Romboutsia* is involved in the production of butyrate (one of the SCFAs) [56] which can help prevent T1D in humans by suppressing the activity of autoimmune T cells [57,58]. SCFAs, including butyrate, can stimulate intestinal L cells to secrete GLP-1, which in turn influences insulin release and pancreatic function [59]. This study indicates that a diabetes-associated shift in the gut microbiota may attenuate bile acid- and SCFA-mediated metabolic and anti-inflammatory signaling, potentially contributing to metabolic dysregulation and low-grade inflammation. Accordingly, strategies aimed at restoring these microbiota-linked functions may be clinically relevant for the management of diabetic dogs. Overall, the gut microbiota changes observed in diabetic dogs are broadly consistent with those from humans, yet notable differences also exist. Taxa that are frequently reported to be significantly altered in human diabetes, such as *P. copri*, as well as putative beneficial genera including *Blautia* and *Roseburia*, did not differ significantly between diabetic and healthy dogs in our cohort. Notably, at the species level, we identified several taxa that have been rarely reported in previous diabetes-related microbiome studies in either dogs or humans, such as *T. bilis*, *Klebsiella variicola*, and *Escherichia albertii*. These findings suggest that canine diabetes may involve host-specific gut microbial features and highlight new candidate taxa for future mechanistic investigations and risk assessment.

Given the marked enrichment of opportunistic-associated taxa and depletion of metabolism-stabilizing taxa in diabetic dogs, we tested whether functional potential composition paralleled the observed community shifts, providing clues to metabolic and inflammatory mechanisms. KEGG pathway analysis predicted significant differences in the functional gene profiles of gut microbiota between diabetic and healthy dogs, which is consistent with previous studies in dogs [17] and humans [14,60]. Combined with KEGG enrichment analysis, a series of microbial functional pathways involving carbohydrate active transport, as well as bacterial virulence and colonization were significantly upregulated. These results are highly similar to the gut microbiota functional potential remodeling patterns observed in patients with human T1D and T2D [14,61,62,63,64,65]. Notably, multiple pathways implicated in bacterial pathogenicity and environmental adaptation were significantly enriched in diabetic dogs, including bacterial secretion systems, lipopolysaccharide biosynthesis, and biofilm formation, among others. This finding closely aligns with the observed significant increase in opportunistic-associated taxa in the gut microbiota of diabetic dogs. The synergistic enrichment of this set of functional modules indicates an enhanced virulence potential of the gut microbial community [66] and increased potential for stable colonization [67], which may in turn contribute to the onset and progression of chronic inflammation and metabolic dysregulation.

Overall, our study revealed marked shifts in both taxonomic composition and functional potential of the gut microbiota in diabetic dogs, which may contribute to chronic inflammation and insulin resistance and thereby promote diabetes progression. Despite these insights, this study has several limitations. First, residual confounding between diabetic and healthy dogs (e.g., age, breed, body condition score, and diet details) may remain despite efforts to control key variables. Notably, BCS was not routinely recorded in our clinical records, preventing formal adjustment for obesity; future studies should include standardized BCS assessment to better control this confounder. Second, LEfSe analysis may produce false-positive biomarker signals; therefore, future studies are warranted to validate the identified features using multivariable frameworks. Third, the cross-sectional design identifies associations but cannot capture temporal dynamics or establish causality; longitudinal studies are needed to clarify microbiota changes across disease progression. Finally, metagenomic annotation reflects gene-level functional potential and lacks strain-level resolution. Future work should incorporate strain typing, quantitative profiling of AMR/virulence determinants, culture-based isolation, and phenotypic validation to better assess the clinical relevance and potential transmission risk of opportunistic pathogens.

## 5. Conclusions

Through metagenomic analysis, this study systematically revealed compositional and functional potential changes in the gut microbiota of diabetic dogs. The gut microbiota of diabetic dogs showed significant alterations in species composition and functional potential profiles. These changes included a marked increase in opportunistic-associated taxa with notable enrichment of *E. coli*, *K. pneumoniae* and *E. faecalis.* In contrast, there was a significant reduction in beneficial bacteria associated with bile acid and SCFA metabolism, such as *T. sanguinis* and *R. ilealis.* Significant upregulation of pathways included the bacterial secretion system, lipopolysaccharide biosynthesis, flagellar assembly, biofilm formation, and bacterial chemotaxis, suggesting an increased potential for virulence and stable colonization of the gut microbial community. These changes may promote chronic low-grade inflammation and insulin resistance as well as metabolic disturbances and drive the onset and progression of diabetes. This implies that future comprehensive management of canine diabetes should not be limited to traditional blood glucose regulation but also incorporate the assessment and intervention of gut microbiota. Dietary adjustments and targeted restoration of beneficial bacterial functions through prebiotics or probiotics hold promise as novel strategies to delay disease progression. Suppression of virulence expression in opportunistic-associated taxa may further improve metabolic outcomes.

## Figures and Tables

**Table 1:** Characteristics of study participants.

Variable	Diabetic Dogs (*n* = 38)	Healthy Dogs (*n* = 37)	*p*-Value
Age	10 (8, 11)	8 (7.5, 10)	*p* < 0.05
Sex (Female/Male)	22/16	14/23	-
Male (intact), *n* (%)	12 (31.6%)	20 (54.1%)	
Male (neutered), *n* (%)	4 (10.5%)	3 (8.1%)	
Female (intact), *n* (%)	13 (34.2%)	8 (21.6%)	
Female (spayed), *n* (%)	9 (23.7%)	6 (16.2%)	
Breed			-
Poodle	22	18	
Mixed-breed	2	4	
Labrador	4	1	
Corgi	0	4	
Others	10	10	
Blood glucose (mg/dL)	429.9 (378.2, 500.7)	98.3 (90.7, 103.0)	*p* < 0.05

## Data Availability

The metagenomic sequencing data generated in this study have been deposited in the NCBI Sequence Read Archive (SRA) under BioProject accession PRJNA1431711. The detailed study protocol and data analysis plans may be obtained from the corresponding author upon reasonable request for purposes of verification or further research.

## Supplementary Materials

The following supporting information can be downloaded at: https://www.mdpi.com/article/10.3390/ani16060936/s1, Figure S1: Bray-Curtis distance distribution at the genus level; Figure S2: Bray-Curtis distance distribution at the species level; Table S1: Brands of commercial dog foods and major nutrient contents; Table S2: Differential analysis of KEGG level 3 pathways; Table S3: Differential analysis of KEGG KOs; Table S4: KEGG enrichment analysis of gut microbiome functions in diabetic and healthy dogs.
