# Supplementary figures and images for: Shotgun Metagenomics Reveals Gut Microbiome Remodeling with Altered Taxonomic Composition and Functional Potential in Diabetic Dogs
Source: Animals (Basel). 2026 Mar 16;16(6):936. doi: 10.3390/ani16060936 (PMC13023283; doi:10.3390/ani16060936)

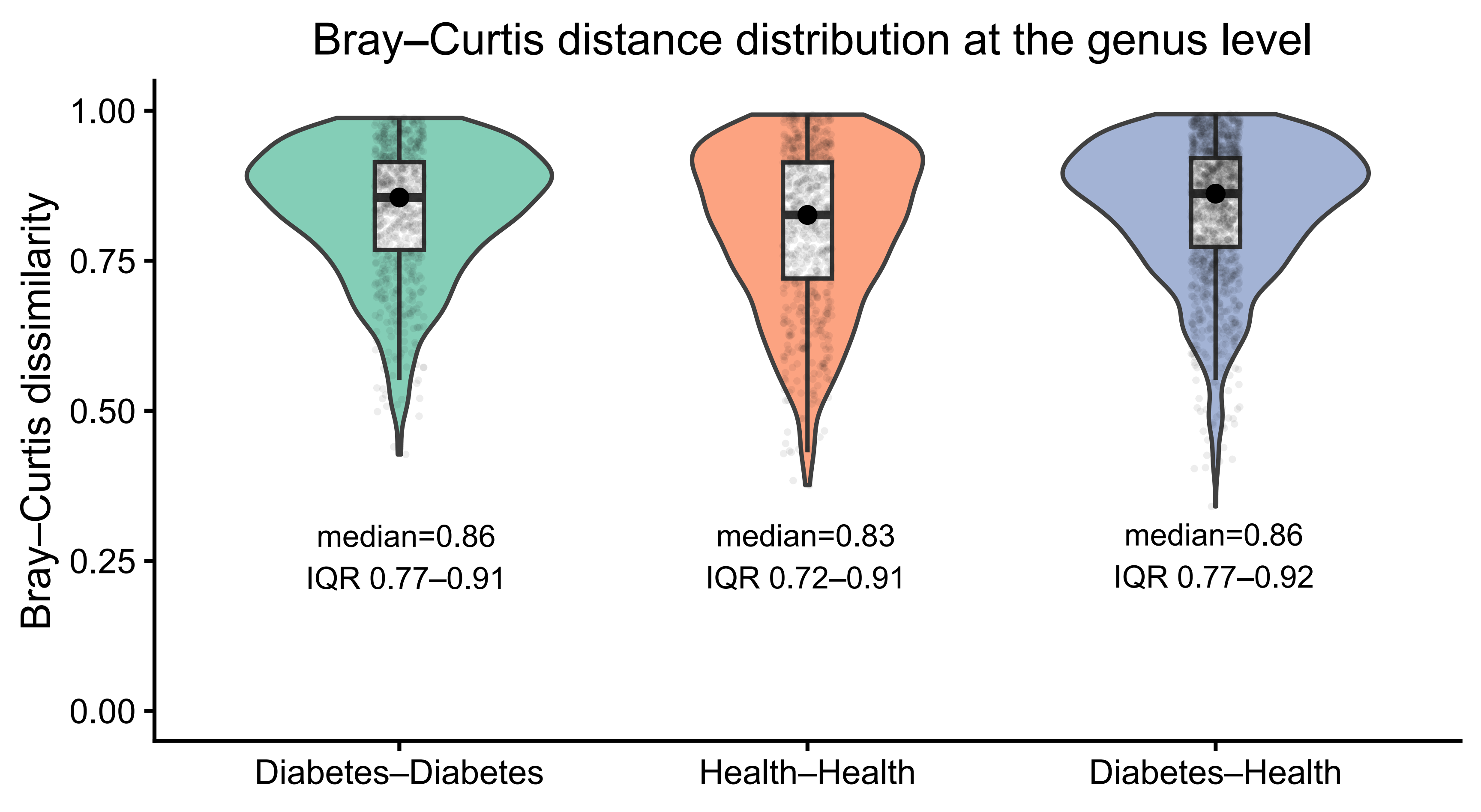

Supplement: Supplementary file 1 [file animals-16-00936-s001.zip › Supplementary Figure S1 Bray-Curtis distance distribution at the genus level.png]

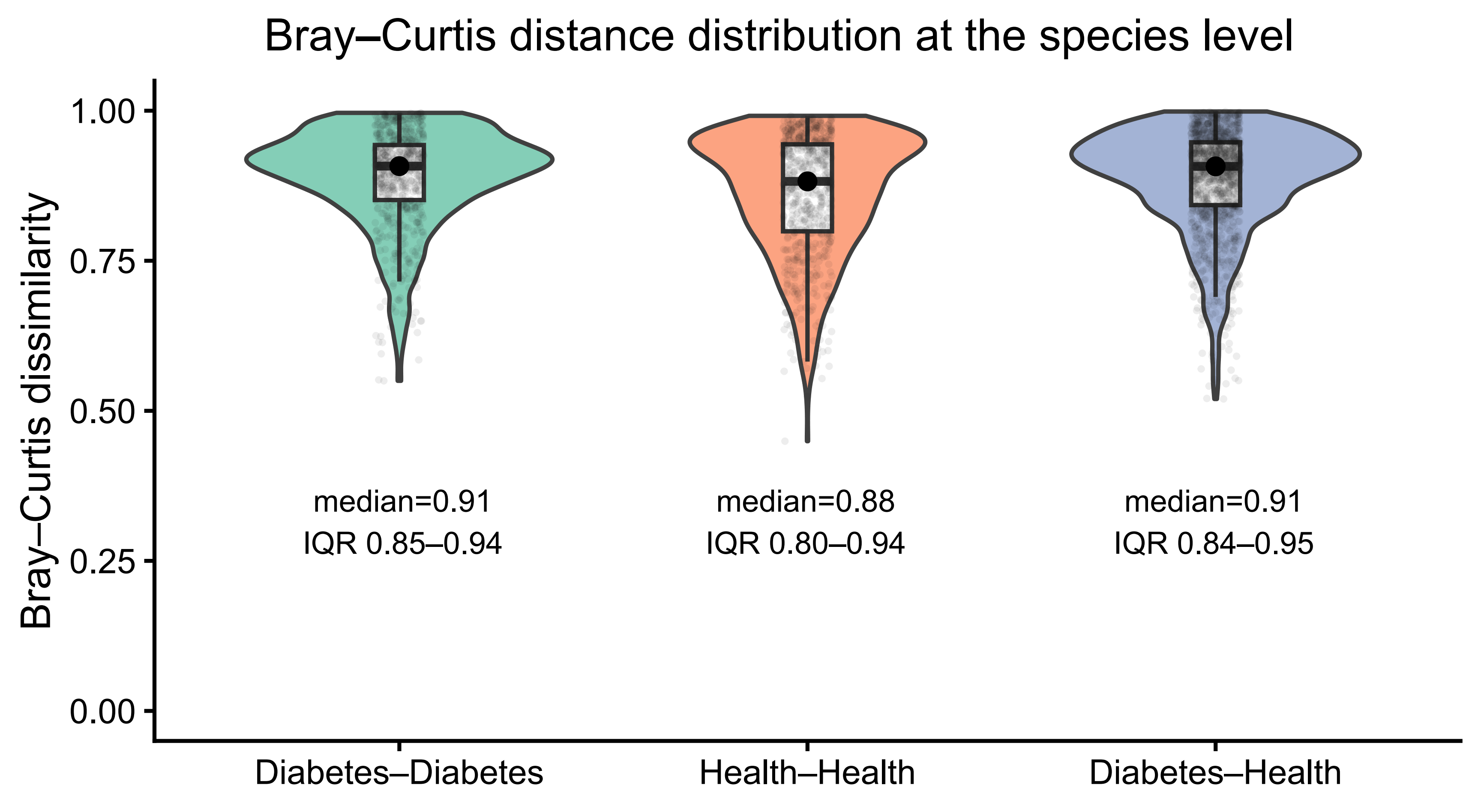

Supplement: Supplementary file 1 [file animals-16-00936-s001.zip › Supplementary Figure S2 Bray-Curtis distance distribution at the species level.png]
